# Monolithic Solder-On Nanoporous Si-Cu Contacts for Stretchable Silicone Composite Sensors

**DOI:** 10.1021/acsami.9b17076

**Published:** 2019-12-06

**Authors:** Michael Kasimatis, Estefania Nunez-Bajo, Max Grell, Yasin Cotur, Giandrin Barandun, Ji-Seon Kim, Firat Güder

**Affiliations:** † Department of Bioengineering, Imperial College London, London SW7 2AZ, U.K.; ‡ Department of Physics, Imperial College London, London SW7 2AZ, U.K.

**Keywords:** soft sensors and electronics, stretchable electronics and sensors, flexible devices, wearable sensors, monolithic integration

## Abstract

We report a method of creating solderable, mechanically robust, electrical contacts to interface (soft) silicone-based strain sensors with conventional (hard) solid-state electronics using a nanoporous Si-Cu composite. The Si-based solder-on electrical contact consists of a copper-plated nanoporous Si top surface formed through metal-assisted chemical etching and electroplating and a smooth Si bottom surface that can be covalently bonded onto silicone-based strain sensors through plasma bonding. We investigated the mechanical and electrical properties of the contacts proposed under relevant ranges of mechanical stress for applications in physiological monitoring and rehabilitation. We also produced a series of proof-of-concept devices, including a wearable respiration monitor, leg band for exercise monitoring, and squeeze ball for monitoring rehabilitation of patients with hand injuries or neurological disorders to demonstrate the mechanical robustness and versatility of the technology developed in real-world applications.

## Introduction

Stretchable electronics have produced a large number of mechanical,^1,2^ chemical and biological sensing elements,^3–6^ piezoelectric generators,^7^ batteries,^8^ and even photovoltaics^9^ since their conception in the late 90s. Stretchable devices can conform to curved surfaces (such as the human body) and instantly adapt to dynamic changes in the geometry of the surface they are attached to; hence, they are exceptionally suitable as wearable devices for physiological monitoring and rehabilitation.^10–12^ Stretchable strain sensors constructed using electrically conductive and elastomeric polymer composites (CPC) can reversibly transduce mechanical deformations into an electrical signal through changes in their resistance or capacitance. Silicone-based stretchable CPC strain sensors are particularly easy to fabricate (in a standard laboratory environment, that is, without the need for advanced facilities such as a cleanroom) and consist of conductive particle fillers dispersed in a silicone polymer matrix to measure applied strain. The sensors can be molded into the shapes desired using 3D-printed molds or printed directly at room temperature via screen or stencil printing.^13,14^ Among others, carbon black-filled polydimethylsiloxane (CB-PDMS) composites are widely used as strain sensors because of the long-term chemical and mechanical stability of silicones, tunable mechanical/electrical properties of these composites, and their low cost.

Works reported to date have optimized the sensing properties and the manufacturing of stretchable CB-PDMS sensing elements; however, reliable interfacial adhesion of the stretchable sensing element with electronic components, such as wiring, integrated circuits, or other conventional devices, still remains a problem.^15–17^ Currently, the electrical connection between CB-PDMS sensors and external electronics primarily relies on brittle conductive adhesives, although some commercial products use large solid mechanical connections, such as clamps and clasps, that are not suitable for miniaturized devices (Smart Garments by Stretchsense, Zephyr Biomodule Device).^18^ Silver-based conductive epoxy (AgEpoxy) is, by far, the most widely used adhesive to create electrical contacts between silicone-based CPC sensing elements and electrical read-out devices.^19–26^ Even though AgEpoxy is an excellent electrical conductor (<10^-4^ Ω·cm), it is susceptible to cracking and debonding from the silicone-based stretchable strain sensors and usually the first interface to fail under rates of strain higher than 10 mm/min (2 mm/s is physiologically relevant).^27^ This is due to the brittle nature of the adhesive and poor wetting of silicones by AgEpoxy. Silicones bond weakly with most adhesives due to their lower surface energy (the surface energy of epoxy is 40-50 mJ/m^2^, while silicones have a range between 19 and 25 mJ/m^2^). Hence, a reliable method is needed for bonding electronic components with silicones.^28^

To overcome the limitations of AgEpoxy, several groups explored vacuum deposition of metallic structures on silicone elastomers and subsequent soldering of wiring and components to achieve bonding.^29–31^ Vacuum deposition of metals on silicones, however, requires complex processes that generally involve patterning through photolithography, deposition of additional adhesion enhancement layers, and liquidphase transfer of films. Despite the complex fabrication methodologies, such metallic contacts are still susceptible to delamination and failure, especially in applications (such as physiological monitoring) where the contacts may experience high levels of applied stress (>1 MPa) and high rates of strain (>2 mm/s).^32,33^

In this work, we propose a method of creating solderable, mechanically robust, electrical contacts to interface (soft) CB-PDMS strain sensors with conventional solid-state (hard) electronics using a nanoporous Si-Cu composite. The Si-based solder-on electrical contact consists of a copper-plated nanoporous Si top surface formed through metal-assisted chemical etching (MACE) and electroplating and a smooth Si bottom surface that can be covalently bonded on silicone-based CPC through one-step plasma bonding. We investigated the mechanical and electrical properties of the contacts proposed under relevant ranges of mechanical stress for applications in physiological monitoring and rehabilitation.

## Results and Discussion

### Fabrication of CB-PDMS and Layered Composite

**Figure 1:**
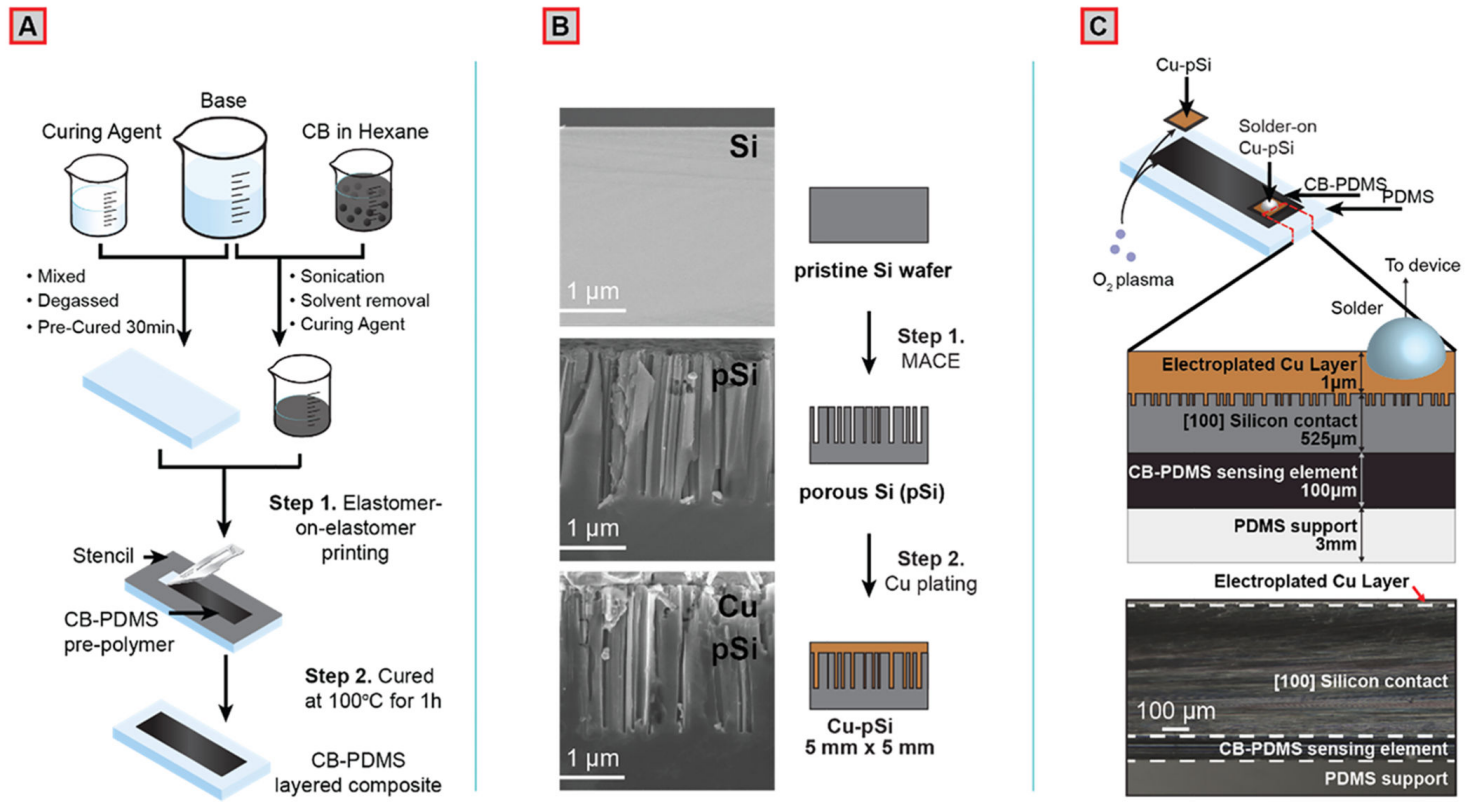
(A) Schematic representation of the fabrication of the stretchable CB-PDMS layered composite. (B) Cross-sectional SEM images taken during MACE and Cu electroplating on a p-type Si (100) wafer. (C) Schematic illustration (top) and cross-sectional optical micrograph (bottom) of Cu-pSi contacts after plasma bonding on the CB-PDMS sensing element.

A range of concentrations between 5 and 20% (w/w) CB in PDMS base were prepared through mechanical mixing, sonication, and solvent evaporation (Figure 1A). The CB-PDMS base was mixed with the curing agent, and a 0.1 mm conductive layer was deposited on a carrier PDMS layer through elastomer-on-elastomer printing using a stencil mask. Both materials were then co-cured in a conventional oven at 100 °C for 1 h (see Supporting Information, Figure S1 for photographs of the printed and co-cured devices).^34^ We fabricated piezoresistive-type strain sensors because they are easily implemented into electrical read-out systems. The fabrication process developed, however, is versatile and can be adapted to different geometries and sensing modalities, including piezocapacitive sensors, and multilayer structures. We characterized the electrical resistance of the unstrained CB-PDMS layer using a commercially available (Uni-Trend Technology Co., Ltd., UT171B) multimeter (current measurement with 5 V applied voltage) and flat alligator clips, with a CB concentration range between 5 and 20% (w/w) of PDMS (Figure S2). The electrical resistance of CB-PDMS decreased rapidly with increasing concentration of CB particles from 9.5 ± 1.5 MΩ for 5% to 4.1 ± 0.3 kΩ for 20% CB due to the formation of conductive percolative networks within the polymer matrix, reaching values around 200 kΩ for 12% CB.^35^

### Si-Based Solder-On Electrical Contacts for Silicone-Based CPC Sensors

The copper-plated nanoporous silicon (Cu-pSi) contacts were produced in a two-step process (Figure 1B): (i) The top surface of a pristine p-type (100) silicon wafer was cleaned, electroless plated with Ag particles and placed in an etching solution (containing H_2_O_2_ and HF) for 7 min to create a nanoporous Si surface through MACE.^36,37^ The surface resulting from etching consisted of 50-300 nm wide pores with a depth of ∼2.4 μm. (ii) The nanoporous surface was electroplated in a 0.8 M CuSO_4_ aqueous bath versus a Pt wire to create a ∼1 μm Cu film on the nanoporous Si (Cu-pSi) surface that can be soldered to external wiring and components. Elemental analysis by energy-dispersive X-ray spectroscopy (EDX) and close-up electron micrographs show a clear separation between the Si and Cu surfaces for unetched Si. For pSi, however, the Cu layer extends inside the porous surface with decreasing amounts with increasing depth likely due to limitations in mass transfer during electrodeposition (Figure S3). This limitation could be overcome through further optimization of the process parameters or using other electroplating regimens such as pulsed electrodeposition.^38^ The nanostructured surface plays the important role of improving the adhesion of the Cu layer without any additional adhesion enhancement layers on the Si surface by increasing the surface area and morphological complexity to create interlocking structures. We performed tensile testing by soldering a 4 mm thick multicore Cu wire on the electroplated Cu layer on the surfaces of unetched Si and nanoporous Si with varying pore sizes. We did not observe a difference on adhesion strength of the Cu layer on pSi when we increased the pore size since, in each experiment, the Si substrate failed before the Cu film could detach from the surface. The unetched Si surface electroplated with Cu, however, delaminated during handling; hence, we were not able to perform tensile testing for these samples (please see Figure S4 for more information).

Electroplated wafers were cut into 5 × 5 mm^2^ pieces by laser, and the unmodified side of each piece (smooth, nonplated, nonporous side of the starting Si wafer) was plasma bonded to the CB-PDMS (Figure S5). Treating the surface of unmodified Si and CB-PDMS with O_2_ plasma for 35 s forms SiO_x_ species on the surface, and when in contact, the materials irreversibly bond together through a condensation reaction.^39^ The multilayered device, including the CB-PDMS sensing element and Cu-pSi solder-on contact, is shown in Figure 1C. Because plasma treatment modifies the mechanical properties of PDMS (increased brittleness; Table S1), a physical mask was used to limit areas of exposure to plasma only at the sites of bonding. AgEpoxy samples were also plasma-treated for equivalence as it is a common industrial method to improve bonding.^40^

### (Electro)Mechanical Characterization of Contact Reliability under Stress

**Figure 2:**
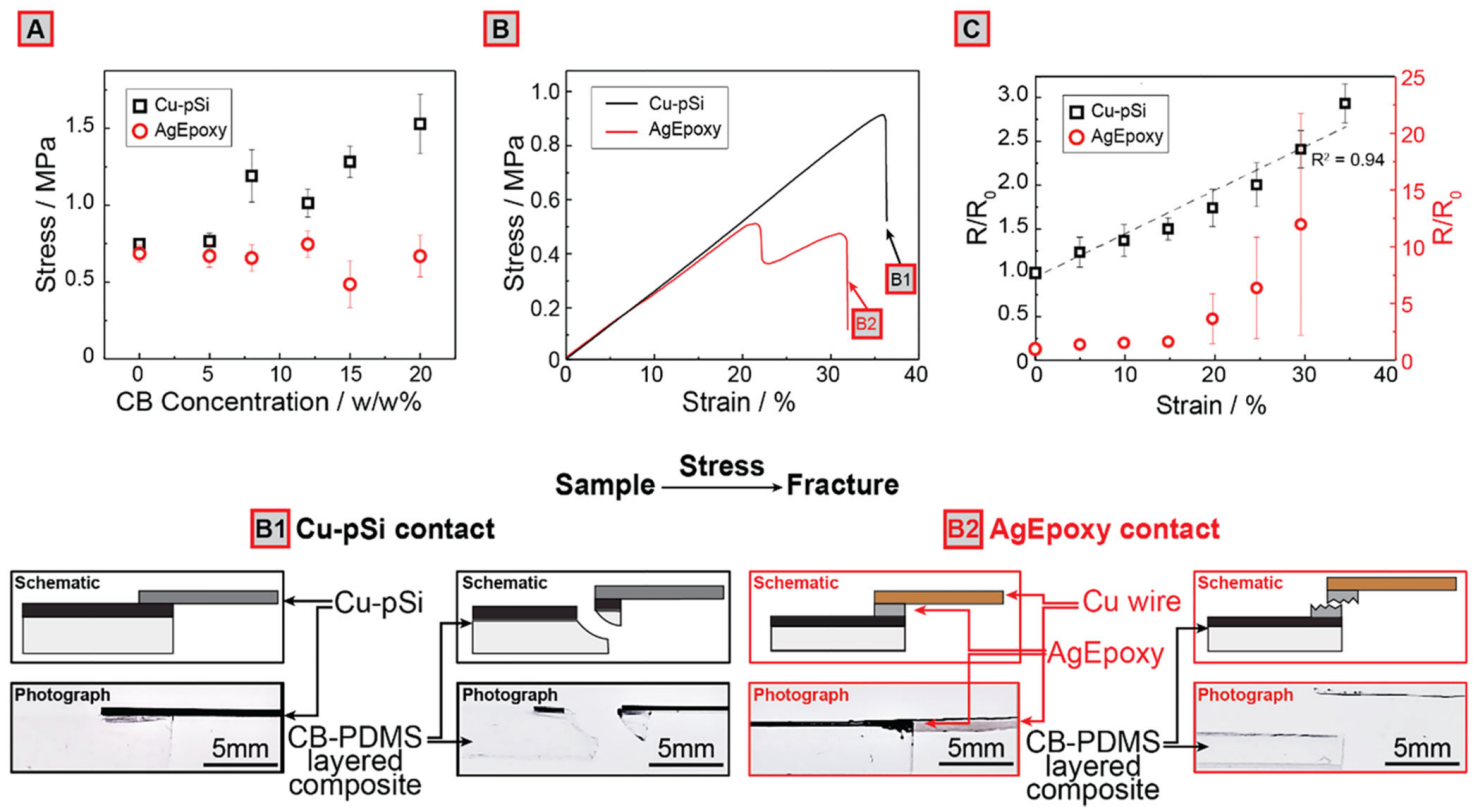
(A) Dependence of maximum stress to failure of Cu-pSi and AgEpoxy contacts in relation to CB filler concentrations (*n* = 5) in the CB-PDMS layer. (B) Representative curves for stress versus strain for CB-PDMS layered composite devices with AgEpoxy and Cu-pSi contacts during a single stretch to failure (*n* = 7 shown in Figure S7). (B1, B2) Modes of failure for 12% CB-PDMS layered composite devices (12% CB content) with AgEpoxy and Cu-pSi contacts used in the experiments shown in (B). (C) Electromechanical characterization of 12% CB-PDMS layered composite devices using different Cu-pSi and AgEpoxy contacts (*n* = 9).

We performed shear strength testing to compare the mechanical characteristics of Cu-pSi and AgEpoxy electrical contacts on the interface of both with the CB-PDMS layered composite (please note that we used AgEpoxy to attach a copper wire to the CB-PDMS layered composite to pull the substrate and make electrical measurements). The samples were stretched from each end until the device-under-test (DUT) failed mechanically (Figure 2). First (Figure 2A), we investigated the effect of CB content (0-20%) on the maximum stress to failure (strain relationship is shown in Figure S6). CB-PDMS layered composite devices fabricated using the Cu-pSi electrical contacts exhibited an increase in maximum stress (n = 5) to failure with increasing CB content in the composite, while the maximum stress to failure for samples with AgEpoxy contacts (n = 5) did not change with increasing CB in the composite conductive layer. Strain stiffening in layered composites can be used in the future to form contact strain relief islands.^41^ This result indicates that each system has a different mode of failure (e.g., failure of the contact-substrate interface, failure of the CB-PDMS layered substrate itself, etc.). To investigate the mode of failure of the CB-PDMS layered composite devices with Cu-pSi and AgEpoxy contacts in more detail, we characterized the mechanical behavior of the devices under shear stress up to 40% strain (Figure 2B and Figure S7). We used CB-PDMS layered composites with 12% CB content for these experiments because it falls in the desired range of electrical resistance (∼200 kΩ; see Figure S2) (a current in the microampere range is desired for further experiments using a 5 V Arduino board). We have discovered that the samples with Cu-pSi contacts produced a highly repeatable and linear strain-stress behavior up to approximately 1.0 MPa (or nearly 35% strain), after which the DUT failed catastrophically with further increases in stress. On the contrary, the CB-PDMS layered composite devices with AgEpoxy contacts exhibited a repeatable and linear stress-strain behavior only up to 0.4 MPa (or 15% strain). Further increases in strain caused devices with AgEpoxy contacts to slowly fail (i.e., no catastrophic failure in this case) as indicated by decreasing stress beyond 15% strain. Visual inspection of the samples before and after shear testing (Figure 2B1,B2) revealed that, in the case of AgEpoxy contacts, the electrical contact-CB-PDMS composite interface failed slowly due to the brittleness of AgEpoxy and noncovalent, weak bonding between the adhesive and silicone substrate. In devices with Cu-pSi contacts, the catastrophic mechanical failure was caused by fracturing of the CB-PDMS layered composite and not the Cu-pSi contact, suggesting that the bonding strength of Cu-pSi contacts was higher than the yield strength of the CB-PDMS layered composite. Electron micrographs of the CB-PDMS layer before and after stretching to 20% showed no cracks on the surface of the composite (Figure S8). We further studied the mechanism of failure of the CB-PDMS layered composite (CB-PDMS on the PDMS carrier substrate) under tensile stress; regardless of the thickness of the PDMS carrier substrate, plasma-treated PDMS always fails around 0.5-0.7 MPa (Figure S9). We speculate that, when subjected to plasma, PDMS becomes more susceptible to forming microscale cracks on its (somewhat hardened) surface. These cracks may subsequently propagate under applied tensile stress (normal to the direction of the crack) and cause failure (i.e., Mode I type crack propagation).^42^ This claim is further supported by the fact that increasing CB content in the CB-PDMS layer also increased the maximum stress to failure (Figure 2A) since composite materials are more resistant to Mode I type crack propagation.^43^ Given that the substrates are flexible, for numerical analysis of the system, a superposition of Mode I and II loading should be considered.

Figure 2C illustrates the electrical resistivity-strain behavior of 12% CB-PDMS layered composite devices connected to read-out electronics via copper wiring soldered on Cu-pSi or adhered with AgEpoxy (see Figure S10). We measured the electromechanical characteristics during a single stretch to failure at a strain rate of 2 mm/s (physiologically relevant rate for breathing).^33^ The resistance response of the CB-PDMS stretchable conductive layer (response = R/R_0_, where R is the measured resistance and R_0_ is the initial or baseline resistance) with Cu-pSi contacts produced a repeatable and slightly nonlinear response up to a maximum strain of 35% (please note that, beyond 35% strain, the devices fail catastrophically). Sensors with AgEpoxy, however, produced a highly nonlinear response across the range of strains between 0 and 30% due to gradual mechanical failure of the AgEpoxy electrical contact beyond 15%. Hence, in all electromechanical tests performed, Cu-pSi appeared to be more robust compared to AgEpoxy electrical contacts for silicone-based CPC.

### Characterization under Cyclic Stress

**Figure 3:**
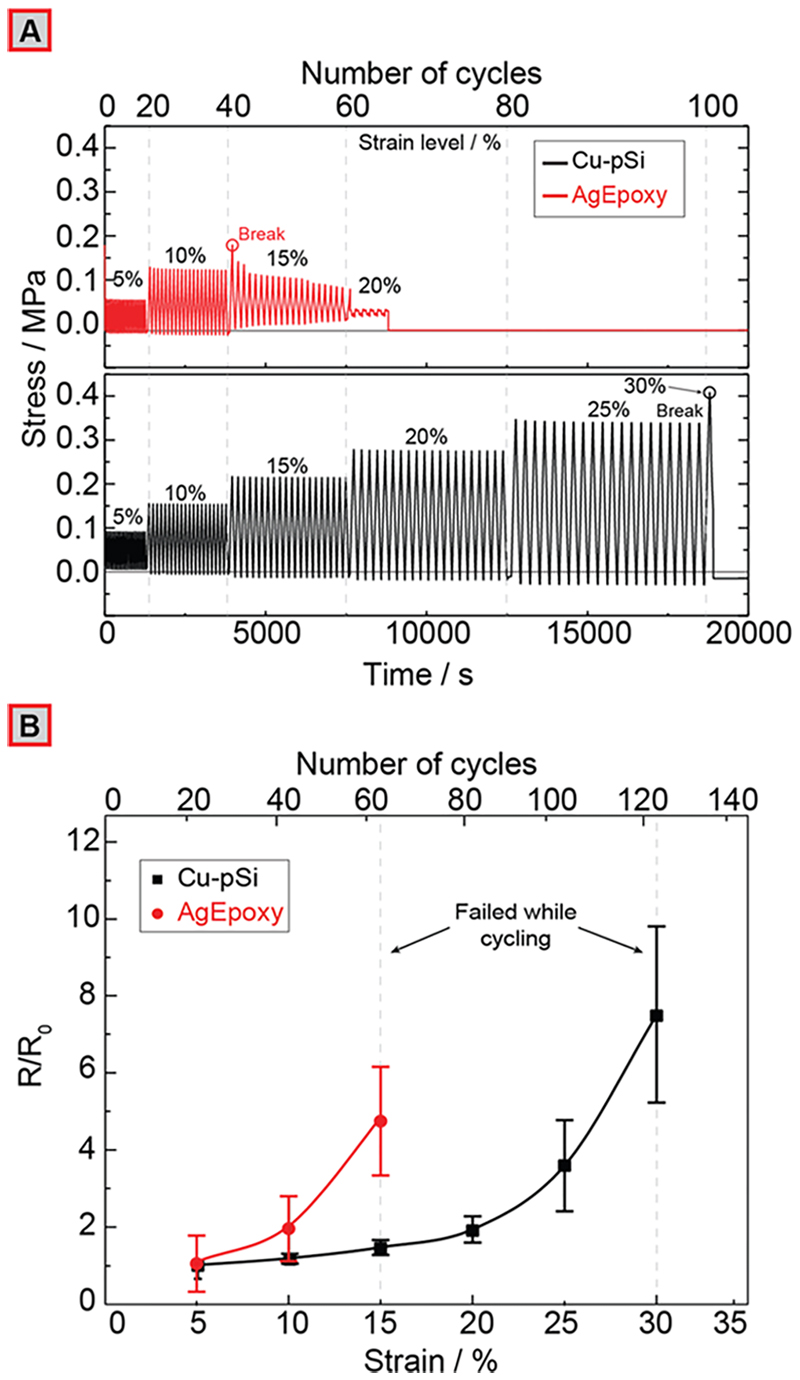
(A) CB-PDMS layered composite with AgEpoxy and Cu-pSi contacts subjected to cyclic strain; AgEpoxy (top) showing gradual failure beyond 15% strain, and Cu-pSi (bottom) showing abrupt failure beyond 30% strain. (B) Electrical resistance after 20 cycles of strain and release at different strain levels (*n* = 5).

Since physiological monitoring (such as breathing) or rehabilitation exercises often involve repeated application of mechanical stress, we also characterized the electromechanical behavior of the CB-PDMS layered composite strain sensors with Cu-pSi and AgEpoxy contacts under cyclic stress. We subjected each sample to 20 cycles of repeated deformation at a strain rate of 2 mm/s at each level of strain starting from 5% to account for stress relaxation in a method modified from Seyedin et al.^44^ After every 20 cycles, the level of strain was increased by 5%. The procedure was repeated until the sample (gradually or catastrophically) failed. Figure 3A shows a representative cyclic stress measurement (see Figure S11 for repeats for n = 5) of the sensors with Cu-pSi and AgEpoxy contacts. Under cyclic stress, the devices with AgEpoxy contacts started failing gradually at strains higher than 10% with complete failure beyond 15%, barely withstanding stress (σ) = 0.2 MPa (we captured gradual cracking and failure of the brittle AgEpoxy contacts and robust stretching of Cu-pSi devices also in a video; see Supplementary Videos V1 and V2). Devices with Cu-pSi contacts, however, would only fail at 30% strain (σ = 0.5 MPa) and remain fully functional under repeated strain until catastrophic failure of the substrate (after about 100 cycles at strains lower than 30%). The electrical measurements under cyclic stress (Figure 3B) also followed a similar trend. At 15% strain, the resistance increased and was eventually lost for the CB-PDMS layered composite devices with AgEpoxy contacts before reaching 20 cycles, whereas the sensors produced with Cu-pSi contacts could reach 30% strain before failing abruptly. The sensors with Cu-pSi contacts, however, produced a nonlinear electrical response with hysteresis especially when subjected to high levels of cyclic stress (>20%) owing to the Mullins effect (i.e., softening due to previous loading) in which the physical connections between the filler (CB) particles are modified to a greater extent when multiple cycles of high levels of strain are applied to the composite material.^45,46^ Since this mechanical effect is prevalent in all in-filled rubbers regardless of the type of contact, we expect that the devices with AgEpoxy contacts would also exhibit a similar behavior of hysteresis at higher strains if they could withstand such levels before failure (see Figure S12 for more information on hysteresis).

### Modeling of an Equivalent Electrical Circuit

**Figure 4:**
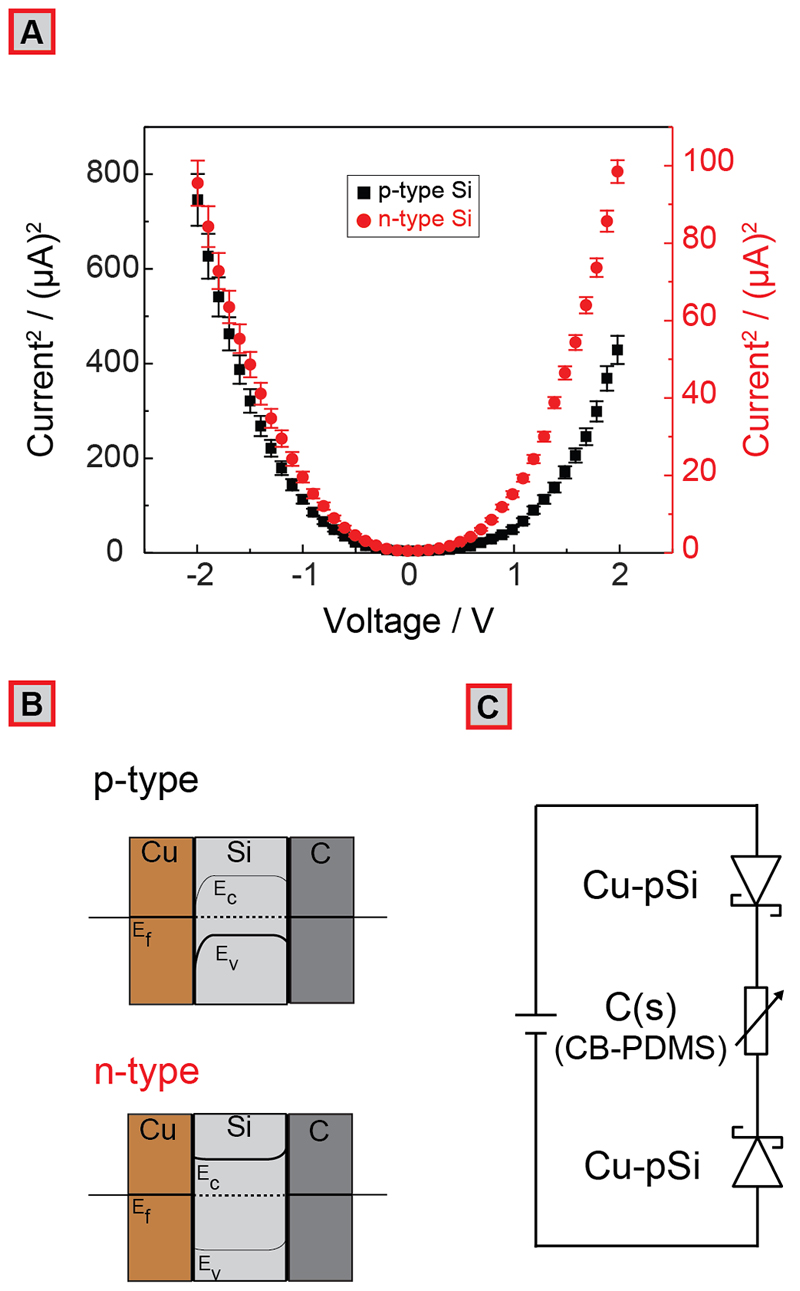
(A) measurements for a 12% CB-PDMS layered composite strain sensor with Cu-pSi contacts created using lightly doped p-type and n-type Si (100) (*n* = 7). Note that for clearer representation, the squared value of the current I is shown in the plot. (B) Equilibrium band diagrams for the Cu-Si-C interface. (C) Equivalent electrical circuit for the CB-PDMS layered strain sensor with two Cu-pSi contacts.

To understand the electrical behavior of the devices formed and to construct an equivalent circuit model, we performed two-probe I-V measurements using CB-PDMS layered composite strain sensors with two Cu-pSi contacts. We also performed four-probe electromechanical tests to determine the contribution of contact resistance to the overall change in resistance under strain. Our results indicate that the Cu-pSi contacts, when attached to the CB-PDMS layer, increase the resistance of the entire device by approximately 100 kΩ at an applied bias (V_D_) of 2 V; this resistance does not change under strain (see Figure S13). The results of the two-probe I-V measurements are shown as I^2^-V in Figure 4A. Because of the metal-semiconductor-metal nature of each electrical junction (i.e., Cu-Si-C), the measurements (n = 7) were highly nonlinear (i.e., non-ohmic). In the range of -2 to 2 V, the devices exhibited a diode-like behavior due to the formation of Schottky barriers (because of the metal-semiconductor interface) with an exponential increase in electrical current for voltages beyond 0.5-1.0 V.^47^ An applied voltage with a magnitude higher than 1.0 V is therefore required for strain sensing with the Cu-Si-C junction. The Cu-Si-C contact structure leads to Schottky junctions at both Si heterointerfaces (Cu-Si and Si-C) for both p-type and n-type Si (equilibrium diagram shown in Figure 4B). Fermi level pinning causes bending in the Si conduction and valence bands, which restricts the conductance at low potentials. The devices constructed using p-type Si, however, were substantially more conductive than the devices created using n-type Si beyond the voltage of operation (i.e., >1 V). This is because the injection barrier height for the metal-Si interface for p-type Si is lower than that of n-type Si, leading to overall larger currents at the same potentials for devices with similar doping levels.^48^ The I^2^-V graph for the junctions constructed using p-type Si indicates more asymmetric barrier properties due to the differences in work functions (Φ_M_) of Cu and C. For example, in the case of p-type Si, because Cu has a work function Φ_M‑Cu_ of 4.6 eV and that for C is 4.8 eV, the barrier height for the Si-Cu junction is larger.^49^ The height of the Schottky barrier can be reduced by using Si with higher doping levels, leading to a higher charge carrier density, thereby reducing the width of the depletion layer. Using thin Si layers (<100 μm) as contacts would also effectively reduce the total width of the depletion layer as the depletion layers for each junction would begin to overlap. An equivalent circuit diagram is presented in Figure 4C, representing the circuit where the CB-PDMS piezoresistive layer is connected via the solder-on Cu-pSi contacts to a power source. Since, regardless of the polarity of the potential applied, one of the junctions is always reverse-biased, and the entire device can be modeled as a variable resistor with two Schottky diodes connected in reverse polarity. Please note that the model proposed is an oversimplification of the system; however, it represents the electrical behavior of the devices measured with sufficient accuracy.

### Demo Devices and Applications

**Figure 5:**
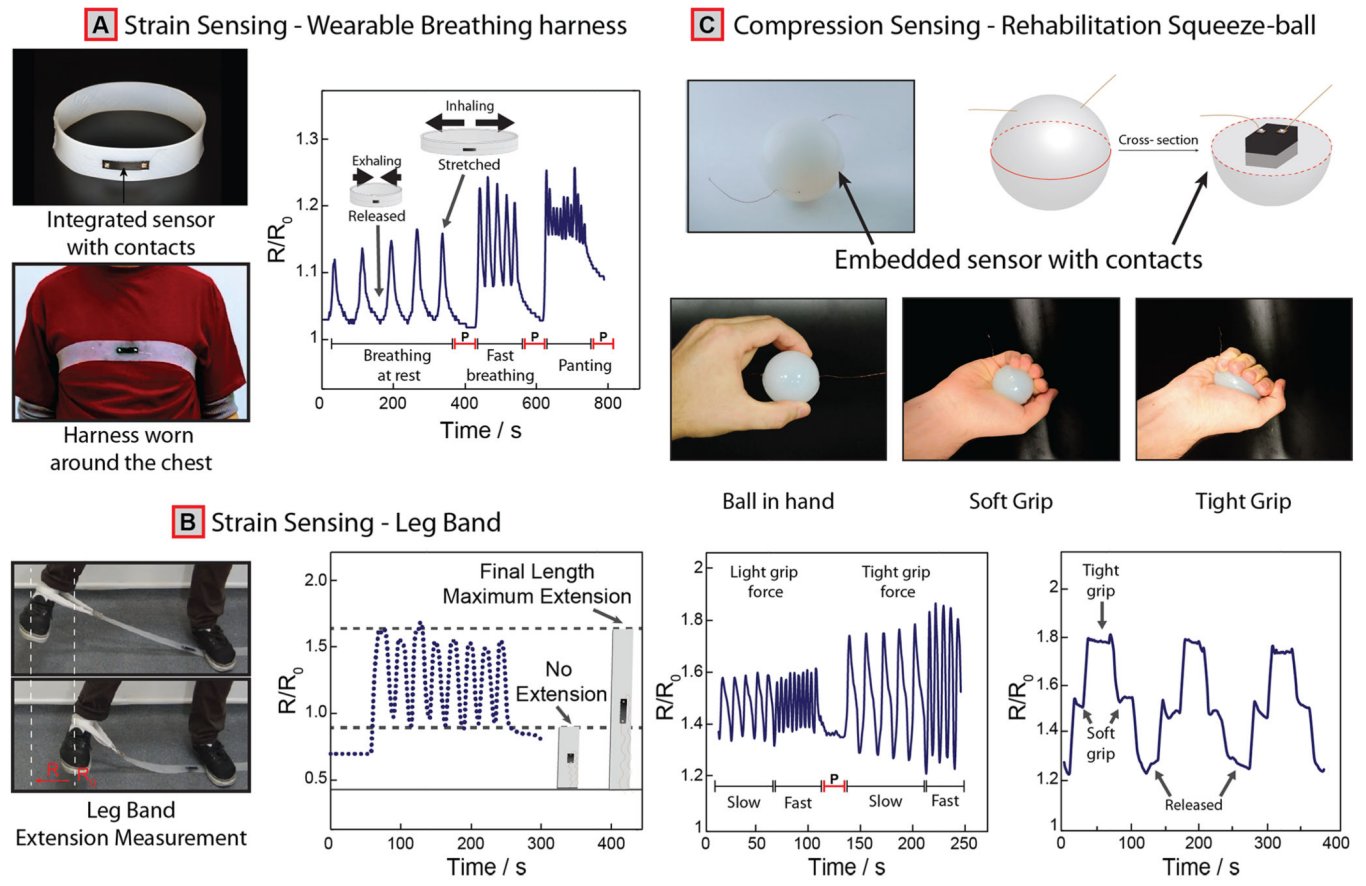
(A) Picture of completed stretchable harness and human subject wearing the device (i.e., CB-PDMS sensor with Cu-pSi contacts); Physiological data collected from the chest during inhalation (stretched) and exhalation (released) recorded with the harness in real time. (B) Picture of a leg band containing the CB-PDMS sensor with Cu-pSi contacts during a leg extension exercise. The electrical signals indicate the level of extension. (C) Picture and graphical illustration of the cross section of the rehabilitation ball and a subject holding the ball for size comparison; resistance data (equivalent to force) collected using the rehabilitation ball during exercise at light and tight gripping force at different exercise rates; testing the sensitivity of the device by applying different grip forces (soft grip → lower force; tight grip → higher force). P = paused.

We have fabricated a series of proof-of-concept devices to demonstrate the mechanical robustness of the technology we developed in real-world applications (Figure 5). In the first example, we printed a CB-PDMS (12% CB) conductive layer with Cu-pSi contacts on an unmodified (nonconductive) silicone-based chest strap (Smooth-On Inc., Ecoflex 00-30 used as the carrier substrate) to measure breathing rate (Supplementary Video V3) to demonstrate the applicability of our technology in wearable physiological monitoring.^5^ Figure 5A shows the change in electrical resistance during inhalation and exhalation due the expansion and contraction of the chest. During inhalation, as the chest expands, the strain sensor stretches, which leads to an increasing in the resistance of the CB-PDMS sensing element. As soon as the exhalation starts, the volume of the chest decreases, leading to a decrease in the electrical resistance. Every peak in the signal corresponds to a cycle of respiratory activity. We were able to monitor breathing at rest (normal breathing), during fast and paused breathing, as well as heavy panting using the device we developed. Other devices, with a similar form factor to the chest strap, can also be produced for various applications in exercise monitoring and rehabilitation such as a leg band (Figure 5B and Supplementary Video V4). In another example, we fabricated a silicone-based, ball-shaped device (i.e., squeeze ball) with embedded CB-PDMS (12% CB) and Cu-pSi contacts for monitoring rehabilitation of patients with hand injuries or neurological disorders such as stroke (Figure 5C). Squeezing the ball compresses the sensing element embedded inside, which in turn changes the resistance relative to the force applied (Supplementary Video V5). Although we did not fully quantify, the sensors were sensitive to both light and tight grip forces and could function reliably for repeated application of force through squeezing of the ball. We have also measured different baseline resistances for the sensing element in a range of physiologically relevant temperatures (Figure S14).

## Conclusions

In conclusion, we have presented a new technology for creating mechanically robust, covalently bonded, Si-based electrical contacts for silicone-based stretchable CPC sensors and electrical devices. Unlike the existing solutions, the technology proposed is reliable, low cost (cost of materials for two 5 × 5 mm^2^ Cu-pSi contacts is $3 × 10^-3^), and simple, consisting of only three steps for the fabrication and attachment of the contacts in a standard laboratory environment, that is, formation of nanoporous Si, electroplating with Cu, and plasma bonding on silicone-based conductive composites. The Cu-pSi contact technology is compatible with large-volume manufacturing through the use of large Si wafers and has the potential to be the enabler of new classes of miniaturized silicone-based commercial stretchable devices with monolithically integrated electrical contacts.^50–53^

One of the most important features of the Cu-pSi technology is that the contacts do not fail gradually (regardless of whether the devices created are subjected to repeated, increasing, or sustained mechanical stress) but instead fail catastrophically. This means that the devices produced using the Cu-pSi technology will either produce a correct signal or do not produce a signal at all; this is especially important for applications in healthcare, for example, disease diagnostics and rehabilitation monitoring, where wrong signals may lead to a different course of medical intervention.

The Cu-pSi contact technology, however, has the following three disadvantages: (i) For the moment, Cu-pSi can only be covalently bonded to silicone-based CPC; therefore, more research is needed for using this solution for other elastomeric materials. (ii) Formation of nanoporous Si using metal-assisted chemical etching requires HF, a highly toxic chemical. This process, however, can be performed with HF solutions with a lower concentration, in line with household cleaning products.^54^ Surface roughening for Si may also be performed using less hazardous compounds such as KOH or potentially even mechanically, eliminating the need for HF. (iii) Si substrates we used in this study were not stretchable nor flexible, but using stress-diffusion geometries and thin layers, Si can be rendered highly flexible and, to some extent, also stretchable. ^55,56^

In the future, the method reported will enable fabrication of highly reliable, battery-powered, portable, wireless, low cost, and integrated sensing systems using silicone-based stretchable sensors. Using a mobile device, such as a smartphone, the data generated by the sensors can be easily processed and stored on the cloud with capabilities for remote access, important for applications in connected/digital healthcare.^5,57^ We believe that the squeeze ball (Figure 5C), in particular, can find immediate applications in the rehabilitation of patients with neurological diseases. Of course, this is just one application, and the method proposed can find other uses such as performance monitoring in athletics and patient monitoring at home or hospitals.

## Experimental Methods

### Preparation of CB-PDMS Composite

A suspension of powdered CB (50 nm particle size, Vulcan XC 72 Cabot Corporation) was mixed with PDMS (Sylgard 184, Corning Inc.) in a five-step process modified from previously reported works:^58–60^ (i) CB was suspended in hexane in a 1:10 (w/w) ratio and sonicated at 220 W for 10 min. (ii) Base elastomer was also dissolved in hexane in a 1:2 (w/w) ratio in a different container. (iii) The CB suspension was mixed with the base solution (from step ii) in different amounts to produce a 5, 8, 10, 12, 15, and 20% (w/w) CB-PDMS precursor. (iv) Dispersions were dried in air overnight to remove the solvent, that is, hexane. (v) The cross-linker was added to form the CB-PDMS composite polymer.

### Preparation of PDMS Carrier Substrate

A PDMS substrate was prepared by mixing degassed base elastomer and cross-linker in a 10:1 ratio (w/w). The mixture was casted on a mold treated with Ambersil PTFE mold release agent and precured at 100 °C for 30 min. As a result, a PDMS carrier substrate with a thickness of 3/6/9 mm was obtained and cut into strips of 10 × 30 mm^2^ using a scalpel.

### Assembly of Strain-Sensitive CB-PDMS Layered Composite (CB-PDMS Composite and PDMS Carrier Substrate)

A plastic stencil with a rectangular pattern of 50 × 5 mm^2^ was cut using a CO_2_ laser cutter (Universal Laser Systems, Inc., model: V460, 60 W). The stencil produced was used to print a 0.1 mm-thick CB-PDMS-based piezoelectric strain-sensitive layer (sensing element) on the PDMS carrier substrate. The device with the printed CB-PDMS pattern was subsequently co-cured at 100 °C for 1 h to form the final structure.

### Preparation of Cu-pSi Contacts

Silicon wafers (p-type and n-type, Siegert Wafer, 525 ± 25 μm thickness, 0-100 Ω·cm, lightly doped) were cleaned with acetone, rinsed with distilled water, and then immersed in a 3:1 piranha solution (95% H_2_SO_4_/30% H_2_O_2_ v/v) at 80 °C to remove organic residues. The wafers were dried in air using a blow dryer; one of the sides was protected with polyimide tape and transferred into a bath containing a solution of 2.9 mM AgNO_3_ for electroless plating of Si with Ag for 2 min to deposit metal particles on the surface. The wafers were rinsed and dried again and immersed in an etching solution of 2.7 M HF with 0.2 M H_2_O_2_ for 7 min to form nanoporous silicon (pSi) by metal-assisted chemical etching (MACE).^36,61,62^ Following etching, the wafers were treated with 10% HNO_3_ to remove all Ag from the surface. Right before electroplating with Cu, each wafer was cleaned one last time with 5% HF to remove the native oxide from the surface of Si and produce an electrically conductive surface. The wafers were plated with Cu in a bath containing an aqueous solution of 0.8 M CuSO_4_ (and 1 mL of ethanol) using a Pt wire as anode. Plating was performed with a current density of 0.2 mA cm^-1^ for 15 min to produce a film of Cu approximately 1 μm. Finally, the wafers were diced into 5 × 5 mm^2^ pieces using a fiber laser (Speedy 100 fiber 20 W, Trotec) to produce Cu-pSi contacts. All chemicals, unless otherwise stated, were purchased from Sigma.

### Attachment of Contacts

#### Cu-pSi

CB-PDMS layers (printed on the PDMS carrier substrate) were protected using a 0.1 mm-thick stencil with 5 × 5 mm^2^ patterns, exposing only the bonding sites. The nonplated side of the Cu-pSi contacts and exposed regions of the CB-PDMS layer were treated with O_2_ plasma (0.4 mbar) for 35 s using a Gala Instruments Plasma Prep 5 Cleaner to prime each element for bonding. The components were bonded together by simply bringing them in contact for 30 s. Tin solder (Sn63-Pb37) was used to solder Cu wires on the Cu-plated side of the Cu-pSi contacts at 300 °C for electrical and mechanical characterization.

#### AgEpoxy

A two-component AgEpoxy mixture (8331S Silver epoxy adhesive, MG Chemicals) is transferred onto the CB-PDMS layers through the same stencil as above using stencil printing. AgEpoxy was dried at 100 °C for at least 2 h to minimize malleability and ensure complete curing.

### Microstructural Characterization

SEM micrographs were acquired using a Zeiss Gemini Sigma 300 FEG SEM at 5 keV electron beam energy. For elemental analysis, we used an Oxford Instruments X-act X-ray detector. Optical micrographs were obtained with a Brunel SP202XM metallurgical microscope. A Nikon D3200 type camera was attached to the microscope, and the images were processed through to a PC in real time using open-source DigiCamControl software to extract scale parameters.

### Electrical and Mechanical Characterization

We used a custom-built circuit (Figure S15) to study the piezoresistive behavior of the devices built during electromechanical testing. This circuit implements a constant current source with optimal temperature stability that renders wearable electronics, such as the breathing harness or squeeze ball, that we manufactured, more reliable outdoors or after prolonged use. The circuit can operate using low voltages and can also be powered by the 3.3 V supply of an Arduino board, which makes it versatile for multiple devices and use cases. The emitter resistor is selected to control the current through the sensing element, and resistance is deduced by measuring the voltage with the known current. We used a second PNP BJT transistor to compensate for voltage drops and temperature fluctuations. The I-V curves for the characterization of the metal-semiconductor interfaces and voltage measurements for four-probe and temperature sensitivity were acquired using a multimeter (Uni-Trend Technology Co., Ltd., UT171B) and a Tenma 72-2535 type variable benchtop DC power supply. Thermal images were recorded using a FLIR E5 thermal imaging camera. Four-probe measurements were performed using a Keithley 6220 precision current source supplying a 3 μA current. Mechanical tests were performed using an Instron 5543 tensile tester (1 kN load cell, Bluehill 3 software). All error bars report standard deviation based on the number of samples reported in the caption.

## Supplementary Material

Supplementary Information
