# Supplementary material for: Monolithic Solder-On Nanoporous Si-Cu Contacts for Stretchable Silicone Composite Sensors
Source: ACS Appl Mater Interfaces. Author manuscript; Available in PMC 2020 Oct 18. (PMC7116211; doi:10.1021/acsami.9b17076)
Supplement: Supplementary Information [file EMS96584-supplement-Supplementary_Information.pdf]

# Supporting Information

## Monolithic Solder-on Nanoporous Si-Cu Contacts for Stretchable Silicone Composite Sensors

*Michael Kasimatis*[1,*], *Estefania Nunez-Bajo*[1], *Max Grell*[1], *Yasin Cotur*[1], *Giandrin Barandun*[1], *Ji-Seon Kim*[2], *Firat Güder*[1,*]

[1]Department of Bioengineering, Imperial College London, London SW7 2AZ, United Kingdom

[2]Department of Physics and Centre for Plastic Electronics, Imperial College London, London SW7 2AZ, United Kingdom

*CORRESPONDING AUTHORS:

M. Kasimatis, e-mail: m.kasimatis16@imperial.ac.uk

F. Güder, e-mail: guder@imperial.ac.uk

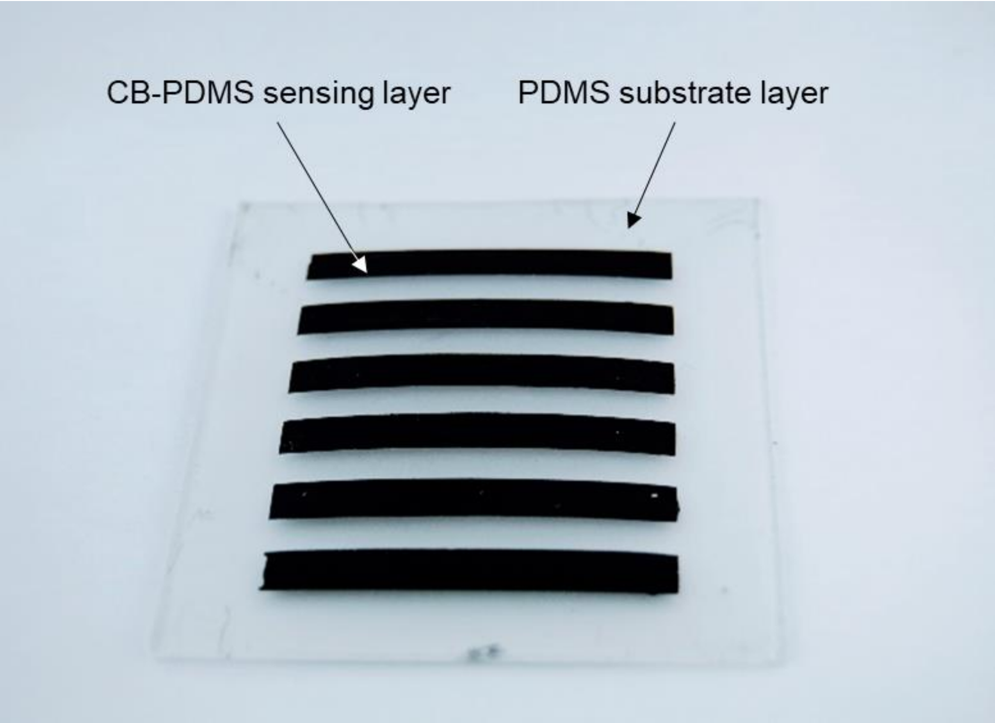


**Figure S1**: Picture of a batch of stencil-printed CB-PDMS sensing elements (5 mm x 60 mm) on PDMS substrate 3 mm thickness.

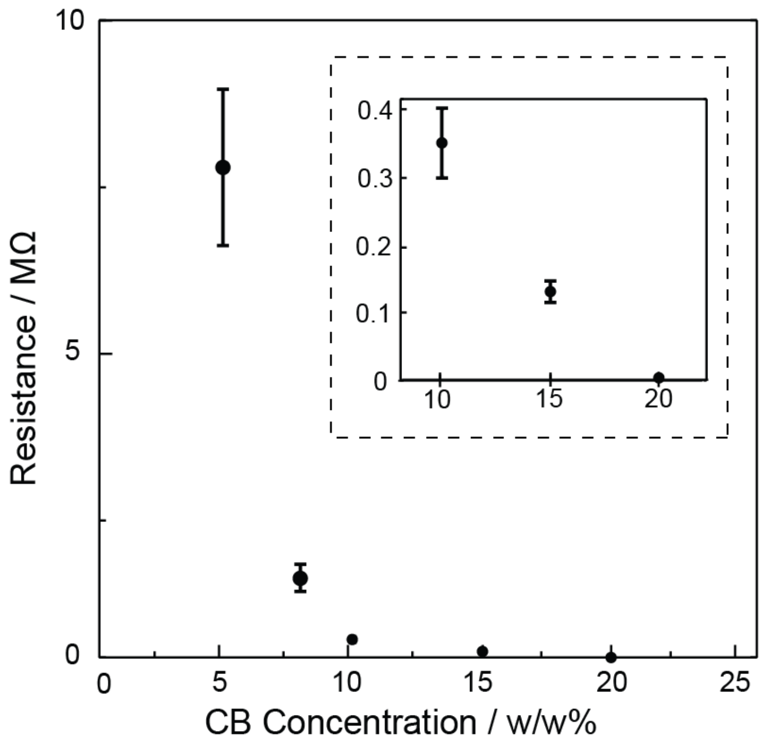


**Figure S2:** Resistance *vs.* CB concentration for the CB-PDMS conductive polymer composite (n=7, error bars represent standard deviation).

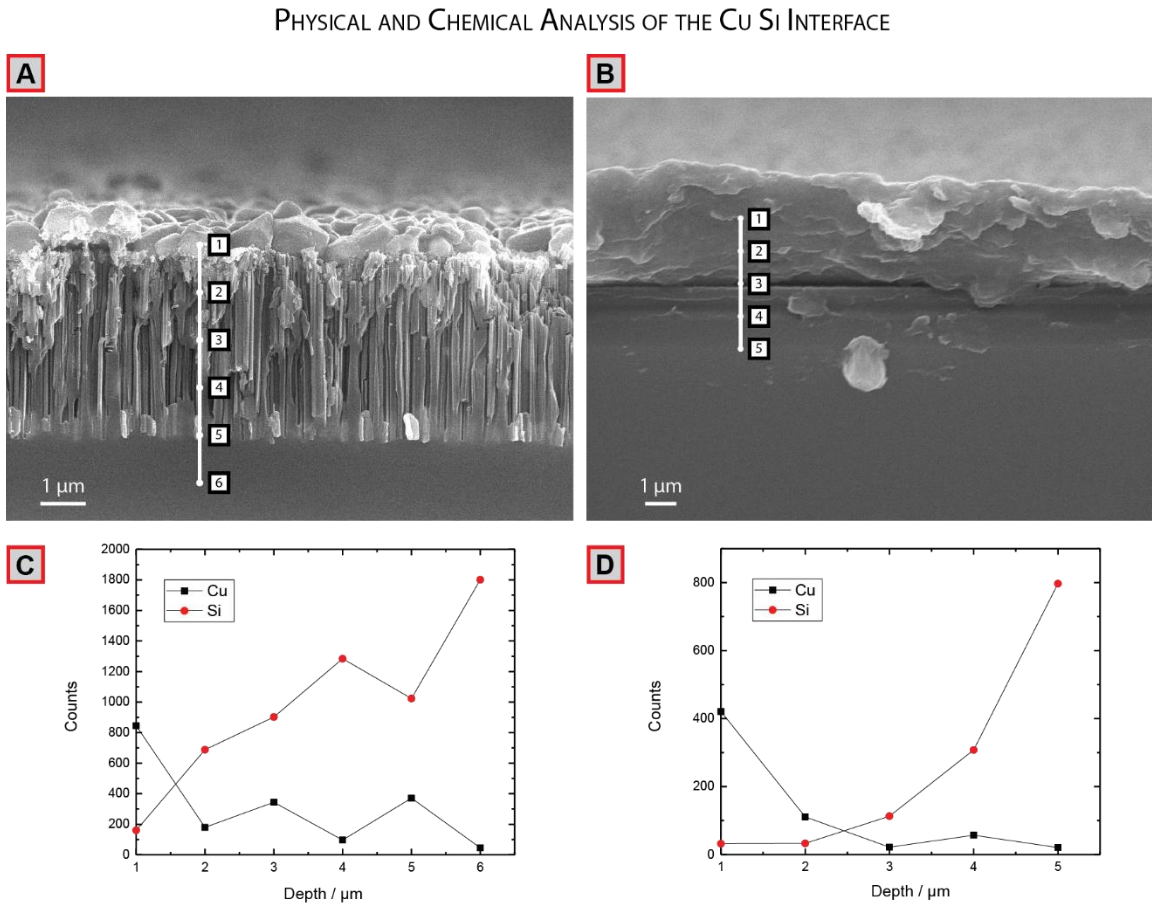


**Figure S3: (A)** Electron micrograph of Cu-pSi interface (Ag deposition time of 2 min). The numbers indicate the location of each spectra acquired by EDX analysis (note that each measurement is 1 μm apart); **(B)** Electron micrograph of unetched Cu-Si interface. The numbers indicate the location of each spectra acquired by EDX analysis (note that each measurement is 1 μm apart); **(C)** EDX spectra for Cu and Si taken from the cross-section of electroplated pSi shown in (A); **(D)** EDX spectra for Cu and Si from the cross-section of electroplated unetched Si shown in (B); Intensity values for Cu and Si were calculated using the peaks at 0.79 keV for Cu and at 1.74 keV for Si.

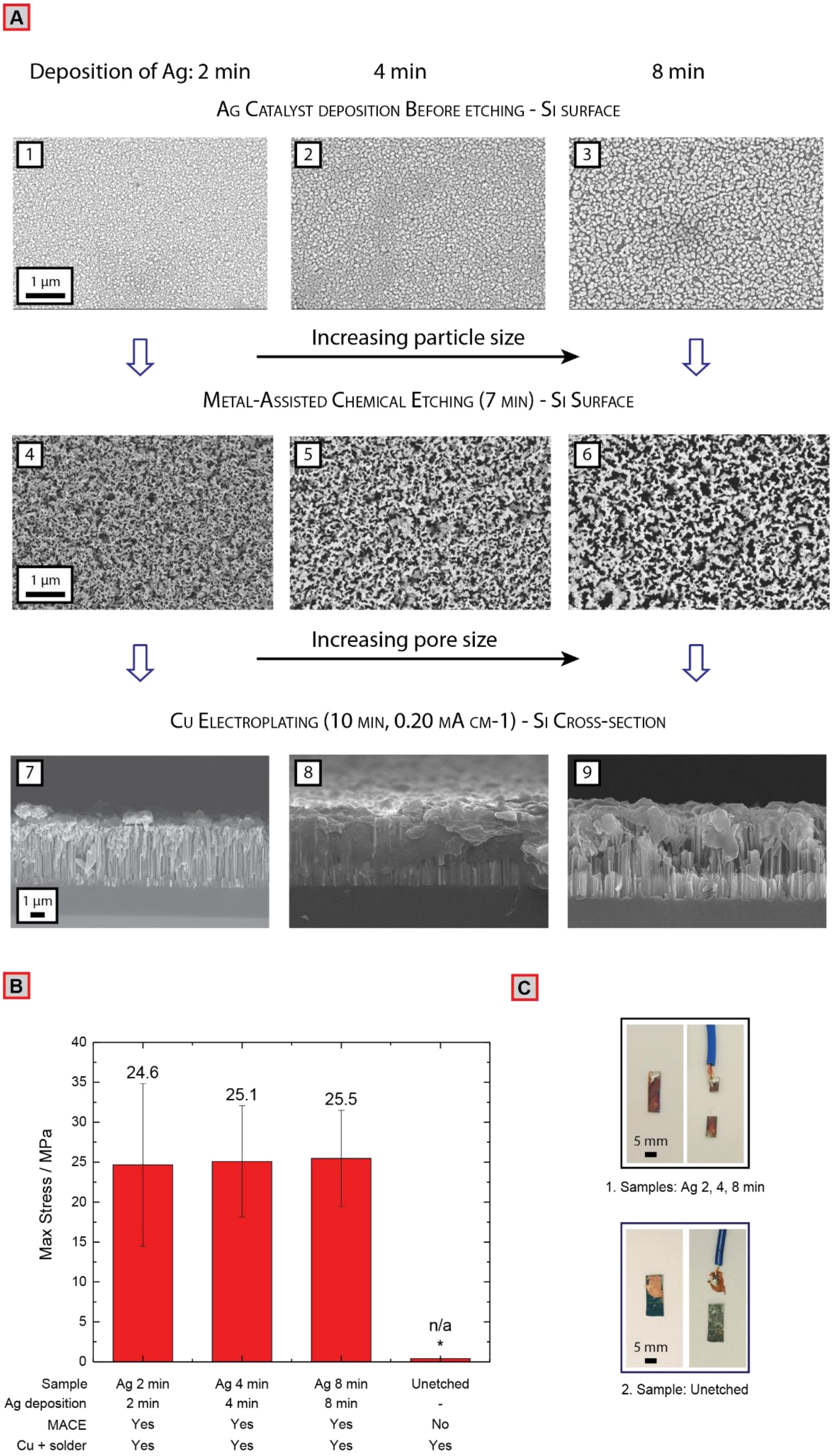
A
Deposition of Ag: 2 min
4 min
8 min
Ag Catalyst deposition Before etching - Si surface
1
2
3
1 µm
Increasing particle size
Metal-Assisted Chemical Etching (7 min) - Si Surface
4
5
6
1 µm
Increasing pore size
Cu Electroplating (10 min, 0.20 mA cm-1) - Si Cross-section
7
8
9
1 µm
B
Max Stress / MPa
40
35
30
25
20
15
10
5
0
24.6
25.1
25.5
n/a
*
Sample
Ag deposition
MACE
Cu + solder
Ag 2 min
2 min
Yes
Yes
Ag 4 min
4 min
Yes
Yes
Ag 8 min
8 min
Yes
Yes
Unetched
-
No
Yes
C
5 mm
1. Samples: Ag 2, 4, 8 min
5 mm
2. Sample: Unetched

**Figure S4: (A1-A3)** Electron micrographs of the surface of Si wafers after depositing Ag catalyst (deposition times: 2 min, 4 min, 8 min for A1, A2, A3 equivalently) with increasing particle size; **(A4-A6**) Electron micrographs of the surface of pSi with increasing Ag catalyst size after etching which produced a Si surface with larger pores; **(A7-A9**) Electron micrographs of pSi with varying pore sizes after Cu electroplating showing the interface of Cu and pSi. The Cu layer was not deposited with an even thickness throughout the cross-section of the pSi, likely due to the limitation in mass transfer during electroplating; **(B)** Max. stress values for Cu-pSi samples pulled to failure after a 4 mm thick multicore copper wire is soldered on the electroplated surface of pSi samples of varying pore sizes and flat, unetched Si. **(C1-C2)** Representative photographs of the samples tested in B showing the wafer before soldering the 4 mm multicore Cu wire (C1-left) and the wafer breaking before the soldered connection (C1-right) and showing the unetched Si wafer just after Cu electroplating (C2-left) and showing the Cu film detaching from the surface of unetched Si during soldering (C2-right). * Max Stress = n/a, unable to perform test; Cu film delaminated during soldering and handling.

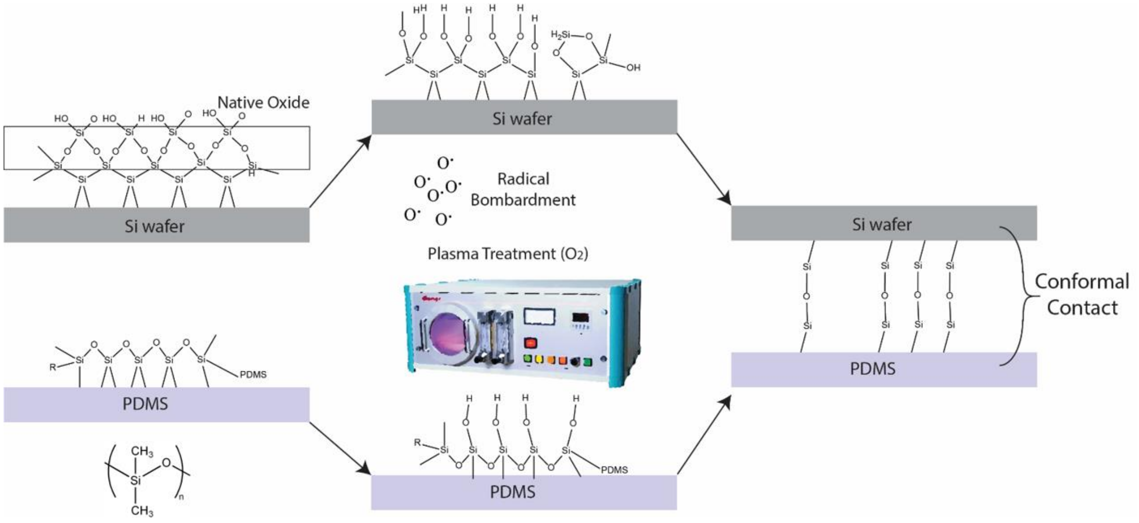


**Figure S5:** Schematic of the plasma bonding condensation reaction between a Si surface (Si wafer) and a silicone surface (PDMS) after coming into conformal contact.

**Table S1**: Stress and strain values for plasma-treated and pristine samples of PDMS (n=5, standard deviation is reported).

| Material | Max Stress (MPa) | Max Strain (mm/mm) | Young's Modulus (hyperelastic region) | Young's Modulus (MPa) |
|---|---|---|---|---|
| **Plasma-treated PDMS** | 1.0 ± 0.1 | 0.8 ± 0.1 | 0.6 ± 0.3 | 1.3 ± 0.6 |
| **Pristine PDMS** | 1.4 ± 0.2 | 1.0 ± 0.2 | 0.8 ± 0.2 | 1.5 ± 0.6 |

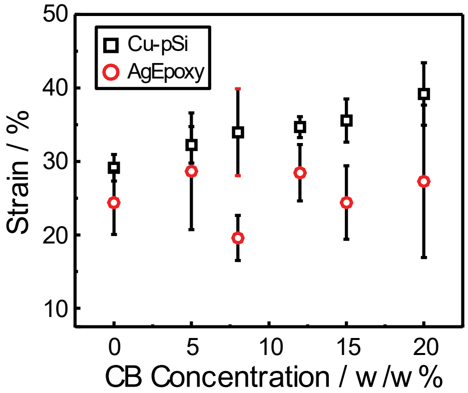


**Figure S6**: Dependence of maximum strain at failure of Cu-pSi and AgEpoxy contacts in relation to CB filler concentrations (n=5) in the CB-PDMS layer.

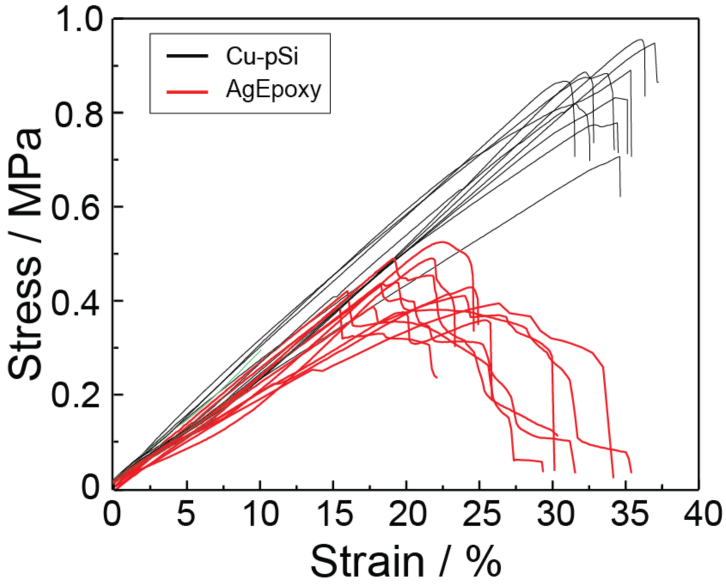


**Figure S7**: Detailed strain – stress characteristics for samples of CB-PDMS (3 mm thickness, 12% CB content) with AgEpoxy and Cu-pSi contacts. The gradual mode of failure for AgEpoxy and abrupt mode of failure (*i.e.* catastrophic) for Cu-pSi samples are shown.

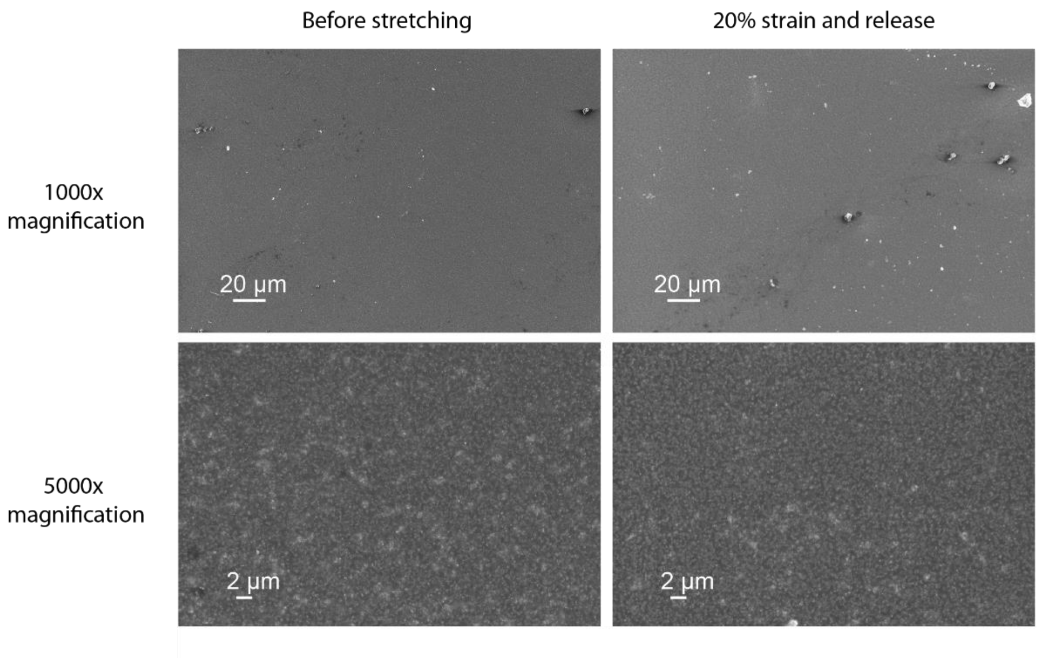


**Figure S8**: SEM micrographs for 12% CB-PDMS surface before and after stretching to 20% strain at 1000x and 5000x magnification.

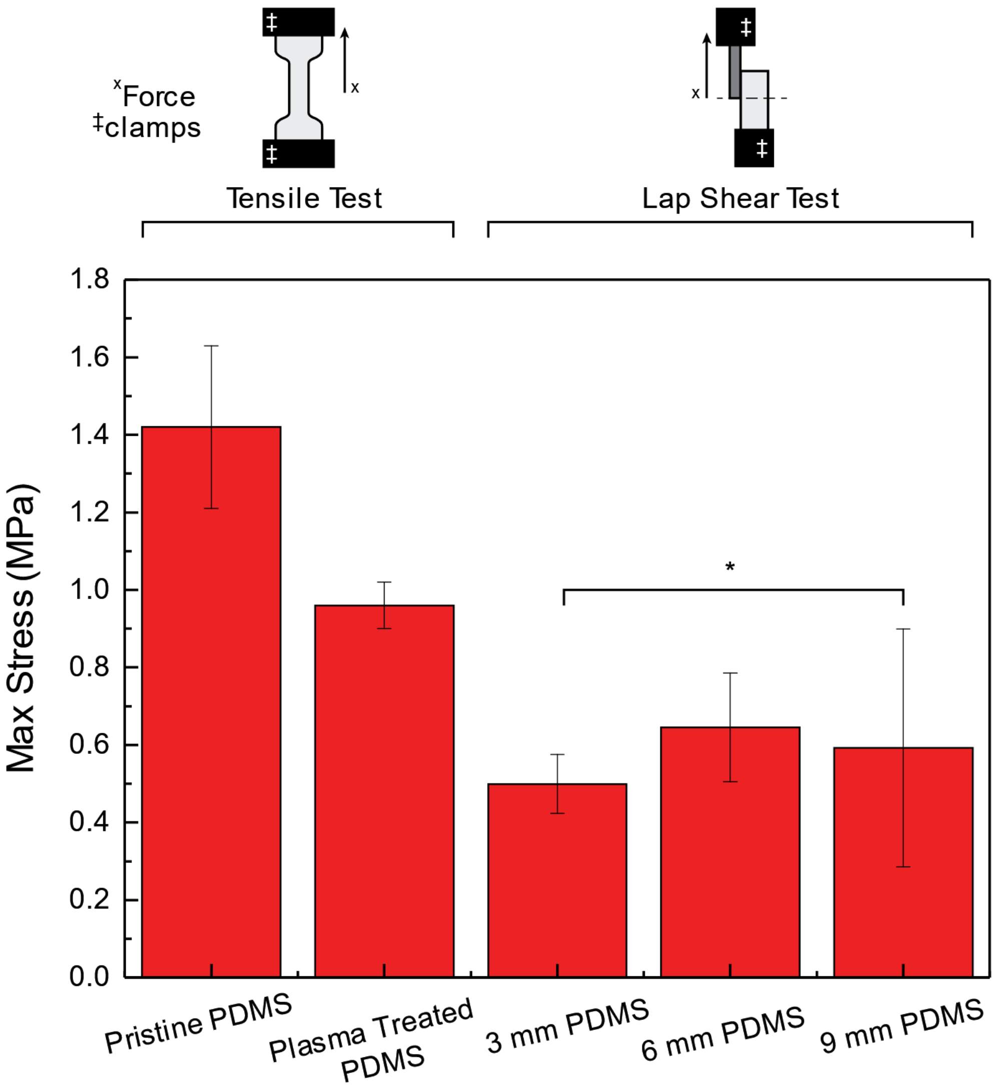


**Figure S9**: Maximum stress experiments using 3 mm pristine PDMS and plasma-treated PDMS (unmasked) dog-bone samples, and samples with Cu-pSi contacts prepared on PDMS substrates with 3, 6 and 9 mm thickness. No difference was observed in the values of maximum stress to failure for each thickness indicating that the fracture mechanism is a surface phenomenon only.

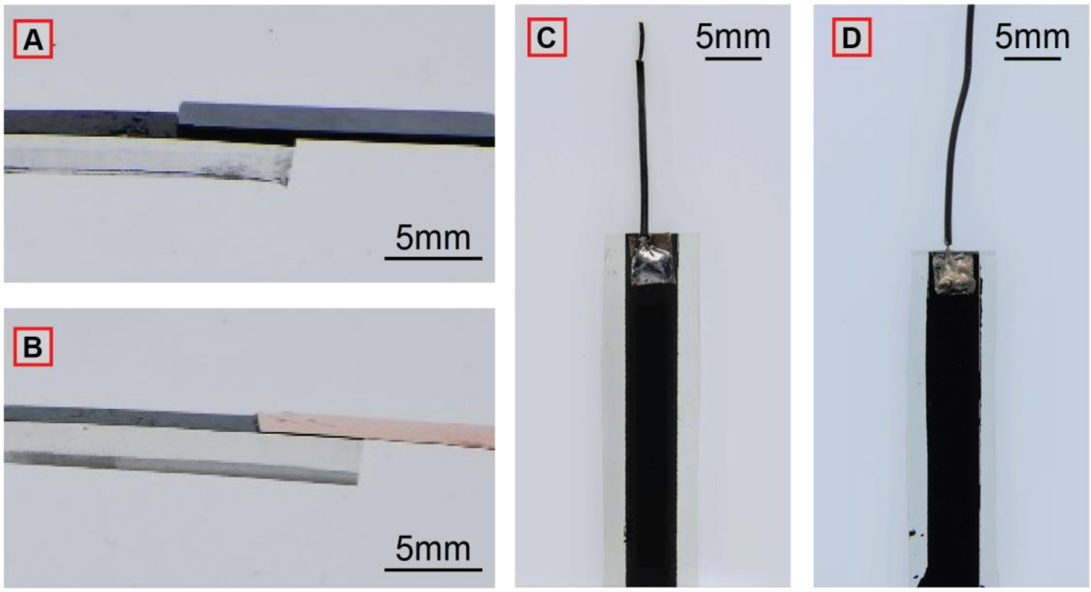


**Figure S10**: **(A, B)** Picture of the samples used in lap shear tests (A - sample with Cu-pSi contact on CB-PDMS; B - sample with Cu wire bonded on with AgEpoxy on CB-PDMS). **(C, D)** Pictures of the assembled sensors with plasma-bonded Cu-pSi contact (C) and stencil-printed AgEpoxy contact (D).

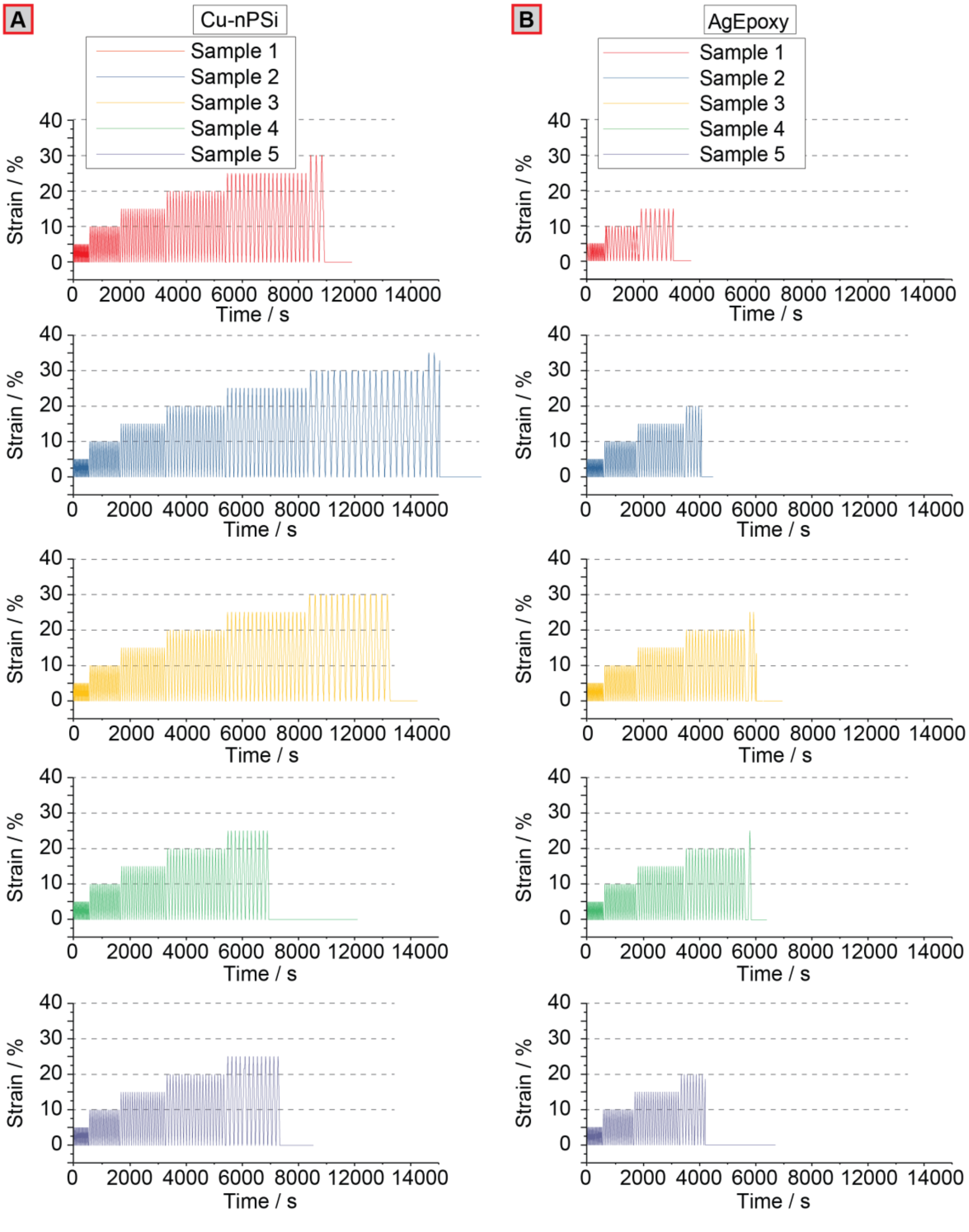


**Figure S11**: **(A)** Strain values for Cu-pSi contact samples during cyclic stretching with increasing strain levels until failure, showing samples failing on average at 30%; during our tests, some samples were able to withstand strains of up to 35% before failure of CB-PDMS; **(B)** Strain values

for AgEpoxy contacts during cyclic stretching at incremental strain levels starting from 5% and increasing by 5% every 20 cycles until failure.

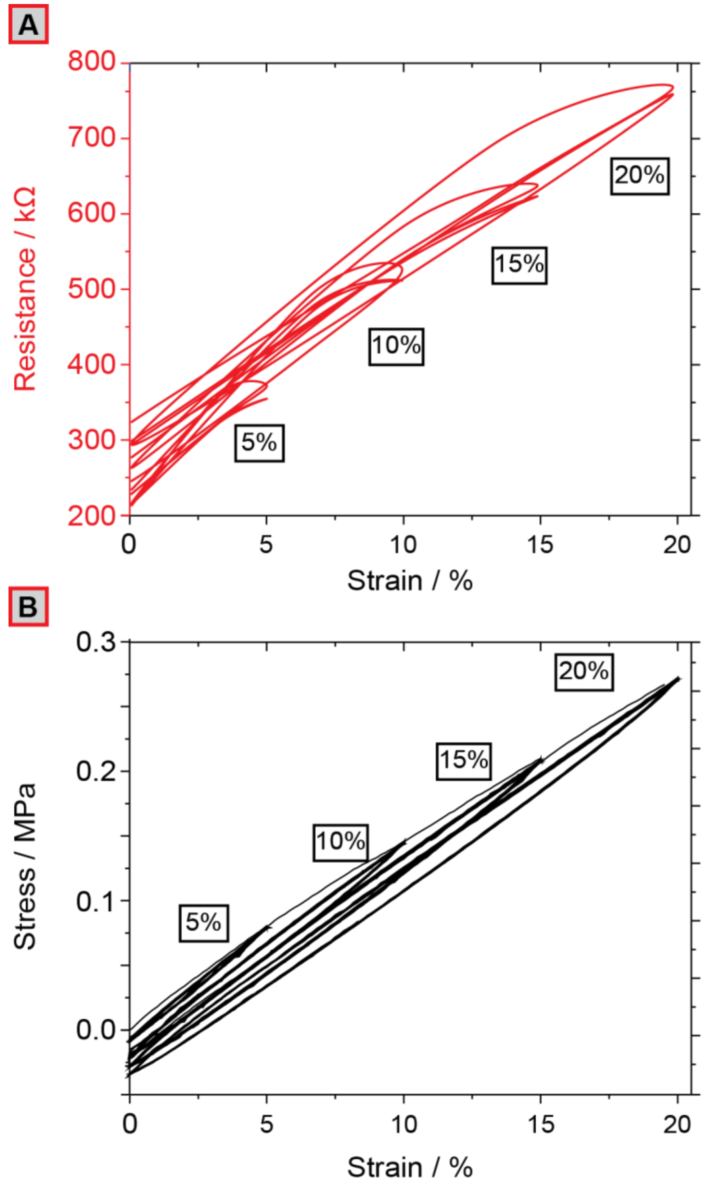


**Figure S12**: **(A)** Representative curves for electrical hysteresis due to previous loading demonstrated on a 12% CB-PDMS layered composite. **(B)** Mechanical hysteresis during cyclic stretching tests.

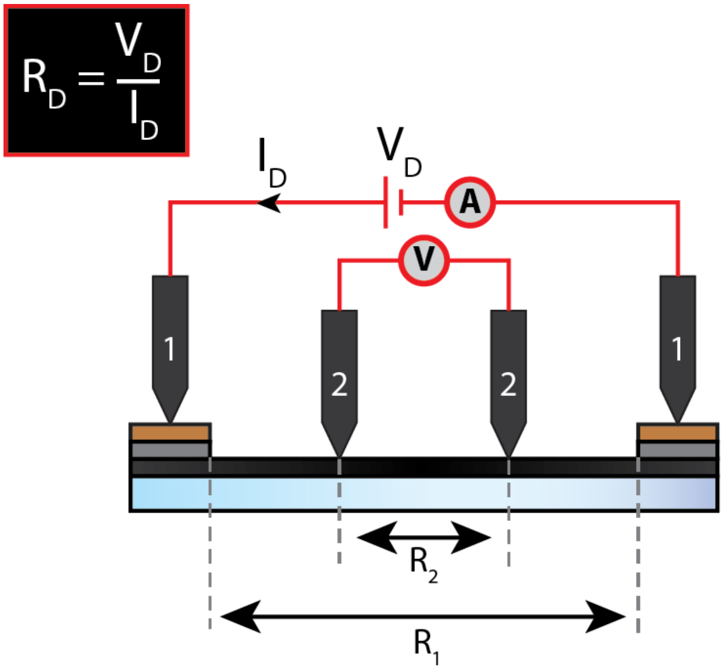


| Strain/ % | $R_D$/ kΩ | $R_2$/ kΩ | $R_1$/ kΩ | Contact contribution $R_D$-$R_1$/ kΩ |
|---|---|---|---|---|
| **0** | 669.7±1.2 | 104.8±3.2 | 564.8±17.5 | 104.8±4.6 |
| **10** | 665.6±0.2 | 99.3±1.1 | 566.2±17.6 | 99.3±4.3 |
| **25** | 675.6±0.3 | 109.1±1.3 | 566.5±17.6 | 109.1±4.8 |

**Figure S13**: Schematic of the four-probe measurement setup to determine the change of contact resistance at different strain levels; **Table**: Derivation of contact resistance contribution for strain levels of 0%, 10%, and 25% (n=3).

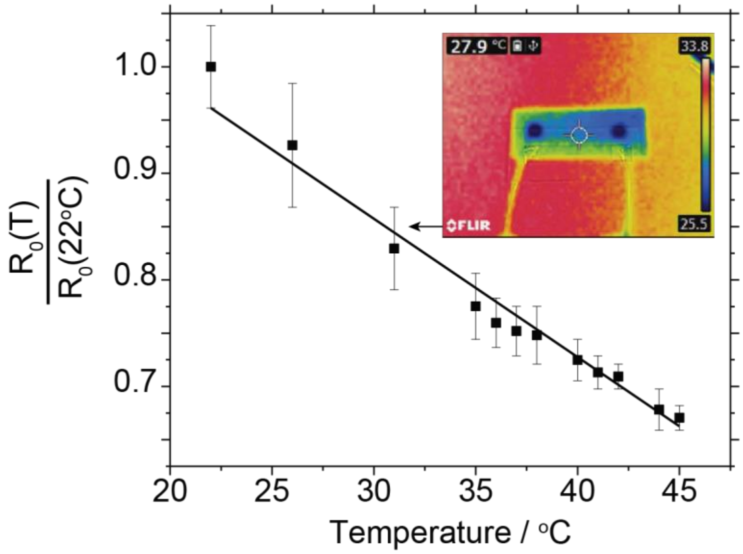


**Figure S14**: Resistance of 12% CB-PDMS layered composite with Cu-pSi contacts as a function of temperature (normalized to resistance at 22°C).

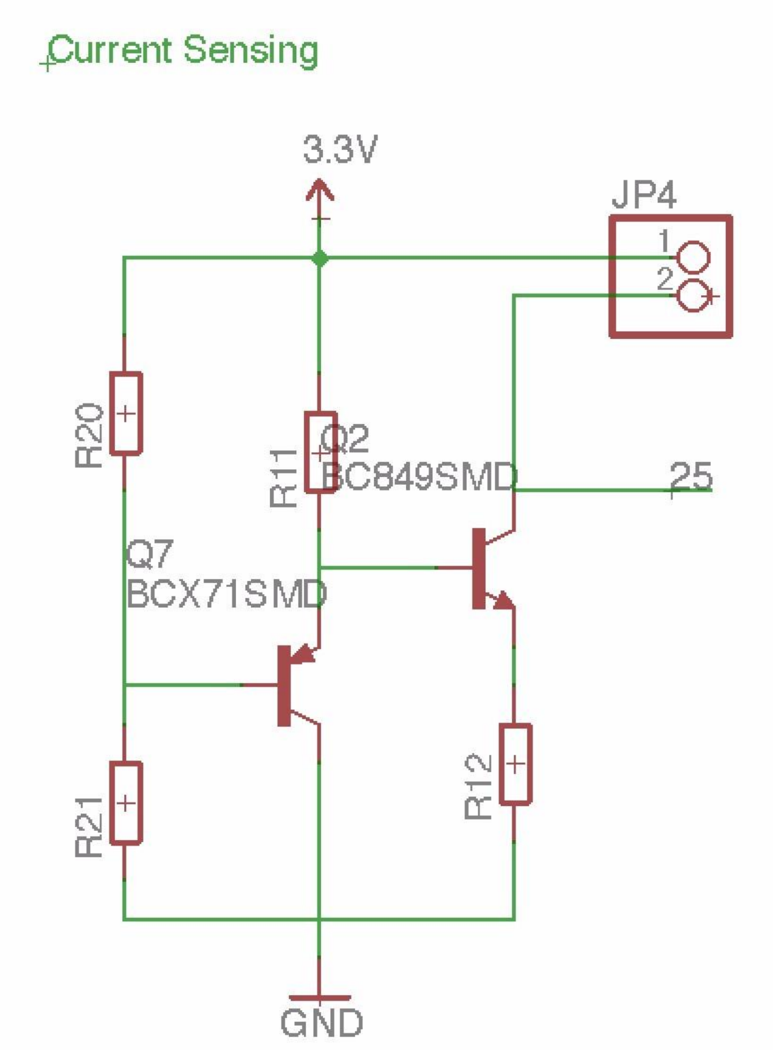


**Figure S15:** Schematic of the custom-built constant current circuit for measuring the resistance of the silicone-based piezoresistive CPC sensors.
